# An ellagitannin-loaded CS-PEG decorated PLGA nano-prototype promotes cell cycle arrest in colorectal cancer cells

**DOI:** 10.1007/s12013-023-01132-5

**Published:** 2023-04-17

**Authors:** Ahmed A. Abd-Rabou, Aziza B. Shalby, Soheir E. Kotob

**Affiliations:** Hormones Department, Medical Research and Clinical Studies Institute, National Research Centre, Dokki, Giza, 12622 Egypt

**Keywords:** Colon cancer cells, PLGA-CS-PEG nanoparticles, Ellagitannins, Chemotherapy

## Abstract

Colorectal cancer is associated with significant morbidity and mortality worldwide. Egypt, as a developing country, has a high-rise incidence of cancer. The current study objective was to investigate the antitumor influences of ellagitannin-loaded CS-PEG-decorated PLGA nano-prototypes against human colorectal cancer cell lines (HCT 116 as well as Caco-2) in vitro. Doxorubicin (DOX), punicalin (PN), and punicalagin (PNG)-encapsulated chitosan-polyethylene glycol-decorated PLGA (PLGA-CS-PEG) nanoparticles (NPs) were described. The cytotoxicity of each preparation was evaluated using MTT assays in HCT 116 as well as Caco-2 cells during G0, G1, S, and G2 cell cycle phases. Cell cycle-related gene expression and protein levels were measured after treatment. Reactive oxygen species (ROS) levels were also measured. Both PN and PNG PLGA-CS-PEG NPs induce colon cancer cell death with cell cycle arrest in the G1 phase in vitro. Caco-2 cells were more sensitive to the nano-therapy than HCT 116 cells. Upon treatment, the ratio of Bax to Bcl-2 expression was increased following nano-therapy, with increased levels of Cas-3 and decreased expression of Bcl-2, PI3k, and NF-ĸB compared to control. The nitric oxide level (NO), a marker of ROS, was increased following nano-therapy compared to control. In conclusion, ROS-mediated cell cycle arrest can be induced by PN as well as PNG nano-therapy in cell lines of colorectal cancer.

## Introduction

The Agency of International for Research on Cancer predicts the global prevalence of colon cancer will increase by 56% of global colon cancer deaths expected to reach 1.6 million deaths by 2040. The greatest increases in cases of colon cancer are predicted in countries with a high Human Development Index [1]. Colorectal cancer (CRC) is the 3^rd^ cancer type that spread between the Americans and Egyptians causing a public health burden. CRC routine screening is currently used to detect early-stage CRC allowing the removal of adenomas and sessile serrated lesions [2].

Mitochondria release cytochrome C which is considered as a pro-apoptotic signal leading to cysteine-aspartic protease family (caspases) activation. Activated caspases act directly to degrade intracellular proteins and nucleus sub-constituents, thereby leading to apoptosis and cell cycle arrest [3]. The PI3K/Akt/NF-kβ signaling pathway was found to play a remarkable action in anti-apoptosis, angiogenesis, as well as metastasis in cells of colon cancer [4]. Accordingly, blockade of PI3K/Akt/NF-kβ signaling pathway may promote cell cycle arrest in colon cancer cells. Recent studies have demonstrated that punicalin (PN) and punicalagin (PNG) nanoparticles promote apoptosis in the cell lines of MCF-7 as well as MDA-MB-231 [5]. The current work, therefore, rolled to assess the efficacy of these NPs on cell cycle arrest in the HCT 116 as well as Caco-2 human cell lines of colorectal cancer. Mitochondria possess important roles as endogenous sensors that can lead to arrest of cell cycle and apoptosis promote [6].

A crucial ingredient for the growth of cancer cells is glucose. One of the main cancer cells’ metabolic regulators is the PI3K/Akt/NF-k pathway, which can both activate glycolysis by phosphorylating hexokinase-2 and directly increase cellular glucose absorption by stimulating the glucose transport receptor GLUT1 [7]. In addition, the PI3K/Akt/NF-kβ pathway modulates the production of ROS via direct modification of mitochondrial bioenergetics as well as NADPH oxidases activation [7, 8].

PNG and PN are the main polyphenolic compounds in pomegranate peel and have been shown to have antioxidant, anti-inflammatory, and antitumor activities [5]. PNG and PN can decrease the extent of DNA damage and activate apoptosis in many cancer cell types. Previous studies have demonstrated the therapeutic efficacy of polyphenolic compounds in several types of cells of cancer through Bax, Bcl-2-linked death promoter (*Bcl*-*XL*) up-regulation, and cell cycle proteins and modulating cell proliferation and apoptosis [5, 9, 10].

The nanotechnology revolution has provided new opportunities in the discovery and design of new anticancer agent and methods of overcoming the limitations of current therapeutic agents including short half-life and chemoresistance [5, 11]. The current work goaled to evaluate the anticancer activity of punicalagin and punicalin nano-formulations via the augmentation of ROS-mediated cell cycle arrest beside the PI3K/NF-kβ signaling pathway suppression in cell lines of colorectal cancer (HCT 116 as well as Caco-2).

## Material and methods

### Material

DOX (Doxorubicin, D5220), MTT assay kit (3-(4,5-dimethylthiazol-2-yl)-2,5-diphenyl-2H-tetrazolium bromide, Cat No. 11465007001), PN (punicalin, Cat. No. 67988), PNG (punicalagin, Cat. No. P0023), CS (chitosan, Cat. No. 448869), PLGA (Poly-lactide-glycolide, Cat. No. P2191), and PEG (polyethylene glycol, Cat. No. 1546445) were bought from Sigma-Aldrich (USA). PI3k (Cat. No. ELK9879), NF-kβ (Cat. No. ELK2228), Caspase3 (Cat. No. ELK1527), Bax (Cat. No. ELK1532), Bcl-2 (Cat. No. ELK1524), and MDA (Malondialdehyde, Cat No. ELK8428) were quantitatively detected by ELISA which purchased from (Biotechnological Company of Wuhan, China). Stress markers: NO; nitric oxide and Zn; zinc colormetric kits were purchased from (Bio-Diagnostics company, Egypt) as well as glucose uptake colormetric kit was bought from (Spectrum Diagnostics company, Egypt). RNeasy mini kit (Cat. No. NC9677589) were obtained from Qiagen. cDNA synthesis kit (Cat. No. K2563, SYBR mix (Cat. No. 4344463), and propidium iodide (Cat. No. P1304MP) were obtained from Thermo Fisher Scientific Co. (USA).

### Nano-prototypes synthesis

#### PLGA nanoparticles synthesis

According to the method of oil-in-water single emulsion solvent evaporation, PLGA nanoparticles were synthesized [12] with few adjustments to previously described methods [5]. Briefly, an emulsion of oil-in-water was created by dissolving PLGA polymer (100 mg) in chloroform (3 ml) to prepare a primary emulsion. This is then done though a microtip probe sonicator (VC 505, Vibracell Sonics, Newton, USA) set of energy output at 55 W, for 2 min over a bath of ice. This organic solvent is allowed to evaporate nightly while the emulsion was agitated. The next day, removal of overabundance of PVA was done using an ultracentrifuge (Sorvall Ultraspeed Centrifuge, Kendro, USA) adjusted at 50,602 × *g* and 4 °C for time of 20 min.

#### PLGA NPs with CS as well as PEG decoration

Chitosan-coated PLGA NPs were prepared according to a similar protocol as described above for the synthesis of nanoparticles of PLGA with little alteration to previously described methods [5].

#### PN, PNG, and DOX NPs synthesis

For curative usages, DOX, PN, and PNG were loaded in PLGA alone, PLGA-co-CS, as well as PLGA-co-CS-co-PEG NPs though the same technique reported recently by *Abd-Rabou’s* team [5], through gathering these drugs to the combination before emulsification, with measurable doses.

### Characterization of nanoparticles

#### Dialysis and entrapment efficiency (EE%)

Synthesized NPs of PN, PNG, and DOX were filtered through dialysis tubing to eliminate impurities and free drugs. The concentrations of nano-conjugated drugs were then calculated from values for the total dosages of the drug and unconjugated drug as described previously [5].

To measure the EE (%) of prepared formulations, plotted the PN, PNG, and DOX calibration curves by using serial dilutions of each drug in a microplate reader. The ratio of the drug amount integrated into NPs to the overall drug amount was used to calculate drug entrapment efficiency.

#### Particle size and zeta potential

The PN, PNG mean particle size, and zeta potential [5], as well as DOX nanoparticles have been evaluated using a Zeta-Sizer light scattering instrument (Nano ZS, Malvern Instruments, UK).

#### Transmission electron microscopy (TEM)

By using TEM, all nanoparticles’ morphology has been evaluated (TEM, Philips CM-10, FEI Inc., USA). Formvar-coated copper grids were filled with nano-suspensions (100 g/ml), as well as when the specimen had dried completely, they were dyed with uranyl acetate (2% w/v) (Electron Microscopy Services, National Research Centre, Egypt). For images capturing and analyzing, we used the Soft Imaging Viewer Software as well as Digital Micrograph.

### In vitro studies

#### Cancer cell proliferation as well as maintenance

Human HCT 116 (Cat. No. CCL-247) as well as Caco-2 (Cat. No. HTB-37) cell lines of colorectal cancer were obtained from Egyptian company (VASCERA) which bought them from the American ATCC company. Colorectal cells were cultivated in RPMI-1640 medium with L-glutamine (Cat No. 12-702Q) and Antibiotics: Penicillin-Streptomycin mix (Cat. No. 11690001) were obtained from (Lonza BioScience). This medium was also supplemented with FBS; fetal bovine serum (Cat No. F4135) was obtained from (Sigma Aldrich, USA) and glucose to nourish the cells for propagation. The final media composition was called complete media and was 10% FBS, 88% medium with L-glutamine, 1% glucose, and antibiotics (1%). These cells have been cultivated in a humidified CO_2_ (5%) atmosphere at temperature 37 °C which is suitable for continued propagation.

#### Cytotoxicity assays

By using HCT116 and Caco-2 cells, MTT assay kit was used to examine each group of medication according to the instructions of the manufacturer. 1 × 10^4^ cells/well of HCT116 and Caco-2 cells were grown in 96-well plates. The culture media was supplemented with the tested medicines at specified concentrations. Nano-formulations as well as their free drug counterparts have been tested in the media of the culture in addition to nano-voids (i.e., nano-capsules without loaded medications). MTT (5 mg/ml) resolved in PBS was provided to the cell culture wells after incubation period of 24 hours, then the wells have been subsequently incubated for 4 h at 37 °C. Dimethyl sulfoxide has been applied to resolve water-insoluble dark blue formazan crystals formed by MTT splitting in metabolically active cells. Using a reader of microplate, absorbance at 455 nm was calculated.

Each drug was added to the culture media of HCT 116 as well as Caco-2 cell lines to inspect the corresponding action of treatment on mitochondrial function and turn cell viability. Cells with treated with PN and PNG prototypes and tracking labeled nanoparticles were applied on those cells at serial levels (0, 25, 50, 75, and 100 µM). Similarly, cells were treated with DOX and nano-DOX at varying concentrations (0, 25, 50, 75, and 100 µM).

#### Half inhibitory concentration and fold change calculations

Half-maximal inhibitory concentrations on cell viability (IC_50_) amount were calculated by plotting cell viability against sample concentration utilizing polynomial fitting (software of OriginPro 8). The nano-formulations fold changes were plotted against fold changes in free counterparts as measured in HCT 116 as well as Caco-2 cell lines.

### Biochemical studies

#### Uptake of glucose measurements

The glucose consumption rates of HCT 116, as well as Caco-2 cells, has been assessed following treatment with various NPs with estimated their corresponding free drug by glucose revelation tools (Spectrum, Egypt). Tested cells have been cultivated in plate (96-well) at 1 × 10^4^ cells/well density for 24 h. After 2 h of cell starvation, glucose (5 mM), as well as free drug or NPs, have been supplemented to the media of the culture. The IC_50_ dosage for each NPs was used in this experiment. Afterward of incubation period of 10 min with the detector of glucose, at A450 nm the measurement of absorbance was performed. The rate of glucose consumption in a measuring unit nominated mmol/L has been determined then detected versus the control one.

#### Measurements of stress markers

In culture media from HCT 116 as well as Caco-2 cells, malondialdehyde (MDA) levels by ELISA technique kit and nitric oxide (NO), as well as zinc (Zn) concentrations, have been estimated by colorimetric kits according to the instructions of the manufacturers. The tested cells had cultivated in plates (96-well) at a density of 1 × 10^4^ cells/well for incubation period of 24 h. Then, the free drug or NPs have been provided to the media of the culture. The proposed PN and PNG formulations of IC_50_ values have been employed in this test. By using a microplate reader, optical density was determined at A_550_ nm for all parameters.

#### The reaction of real-time polymerase chain

Colorectal cells’ total RNA has been harvested by the RNeasy mini kits (Qiagen, Germany). cDNA has been synthesized using kits of first-strand cDNA synthesis (Intron Biotechnology, Korea). BCL-2, Bax, Cas3, NF-κB, as well as PI3k genes relative expression levels, have been carried out. A housekeeping gene was GAPDH (forward “F” primer, 5′-GTCTCCTCTGACTTCAACAGCG-3′; and reverse primer “R,” 5′-ACCACCCTGTTGCTGTAGCCAA-3′). A DT-lite Real-Time PCR System (English/Russian system) has been applied to evaluate copy number of cDNA. Reactions of PCR have been established in 25 µL reaction mixtures compressed of 12.5 µL SYBR Premix (Thermo fisher scientific, USA), 0.5 µL 0.2 mM of both F as well as R primers; BCL-2 gene (F, 5′-CTGCACCTGACGCCCTTCACC-3′ and R, 5′-CACATGACCCCACCGAACTCAAAGA-3′); Bax gene (F, 5′-ATGGCTTCTATGAGGCTGAG-3′ and R, 5′-CGGCCCCAGTTGAAGTTG-3′), Cas3 gene (F, 5′-GTGGAACTGACGATGTGGC-3′ and R, 5′-CGCAAAGTGACTGGATGAACC-3′), NF-kβ gene (F, 5′-ATGGCTTCTATGAGGCTGAG-3′ and R, 5′-GTTGTTGTTGGTCTGGATGC-3′), and PI3k gene (F, 5′-GCTCTCGGTTGATTCCAACGT-3′ and R, 5′-ATGGCTTCTATGAGGCTGAG-3′) in addition to 6.5 µL distilled water and 5 µL of cDNA template. The reaction has been performed three steps as follows: 95.0 °C for 3 min; 40 cycles of 95.0 °C for 15 s, 55.0 °C–60.0 °C for 30 s, and 72.0 °C for 30 s; and finally starting at 60.0 °C then increasing by approximately 0.5 °C every 10 s up to 95.0 °C. At the end of each qRT-PCR, a melting curve analysis has been carried out performed at 95.0 °C, then 55.0 °C–60.0 °C, then 95.0 °C for checking the quality of the used primer.

#### Protein measurements using ELISA

Human proteins Bax, BCL-2, Cas3, in addition to PI3K as well as NF-κB were quantitatively measured with commercial ELISA kits (Wuhan Fine Biotech Co., China).

#### Statistical analyses

The repetition of all assays were in a triplicate (*n* = 3). Comparisons between nano-prototypes and the corresponding free drug groups against controls have been performed by SPSS (version 22) through One-Way ANOVA test. In this test, the means of two or more independent groups were compared for detecting whether there was statistical evidence that the accompanied with population means were significantly different. We categorized the significance into three signs. * means that there were statistical variation between the tested counterparts as well as the control (*P* < 0.05). # means that there was high statistical difference between the tested counterparts as well as the control (*P* < 0.01). Finally, non-significance (NS) refers to (*P* > 0.05).

## Results

### NPs characterization

**Fig. 1:**
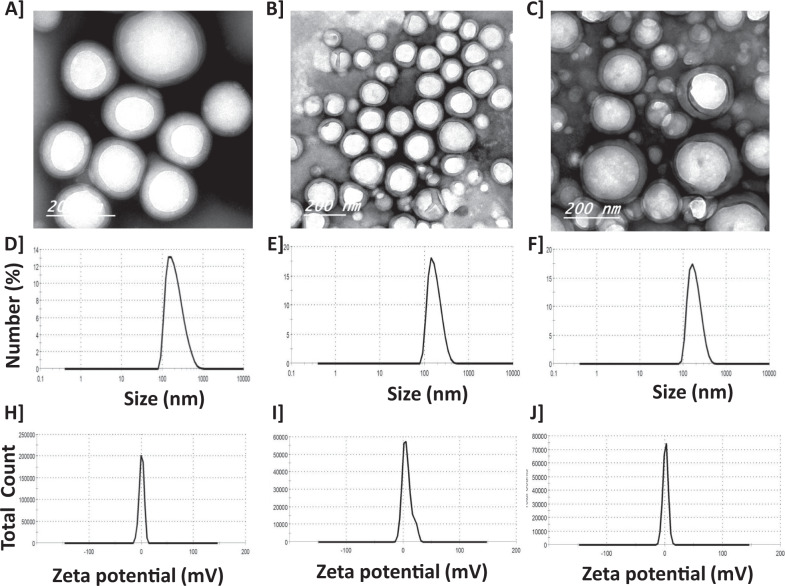
Characterization of DOX PLGA-CS-PEG nanoparticles. Transmssion electron micrscopy (TEM) of DOX PLGA (**A**), DOX PLGA-CS (**B**), and DOX PLGA-CS-PEG (**C**) nanoparticles appearing spherical particles comprising a core with a surrounding capsule. Size distribution of DOX PLGA (221.6 nm, PDI = 0.19) (**D**), DOX PLGA-CS (176.8 nm, PDI = 0.073) (**E**), and DOX PLGA-CS-PEG (192.4 nm, PDI = 0.073) (**F**) nanoparticles. Zeta potential of DOX PLGA (0.663 mV) (**H**), DOX PLGA-CS (6.6 mV) (**I**), and DOX PLGA-CS-PEG (1.3 mV) (**J**) nanoparticles. EE% of DOX inside DOX PLGA, DOX PLGA-CS, and DOX PLGA-CS-PEG are 73%, 77%, and 72%, respectively

The PN and PNG nanoparticles EE%, TEM images, size distribution, and zeta potential and their corresponding nano-voids (i.e., PLGA, PLGA-CS as well as PLGA-CS-PEG nano-voids) were discussed in our recently published paper [5]. In addition, the same characterization parameters for DOX nanoparticles were measured in the present study in (Fig. 1). TEM findings for nanoparticles of DOX PLGA, DOX PLGA-CS, as well as DOX PLGA-CS-PEG are shown in Fig. 1A–C, respectively. TEM images demonstrated that all proposed NPs were spherical in shape and contained a core with a surrounding shell comprised of polymer(s). Furthermore, the mean size of DOX PLGA NPs was 221.6 nm with a poldispersity index of 0.19 (Fig. 1D). The mean size of DOX PLGA-CS NPs was 176.8 nm with a poldispersity index of 0.073 (Fig. 1E). The mean size of DOX PLGA-CS-PEG NPs was 192.4 nm with a poldispersity index of 0.073 (Fig. 1F).

All nanoparticles had positively charged surfaces. The zeta potential of DOX PLGA was +0.663 mV (Fig. 1H), of DOX PLGA-CS was +6.6 mV (Fig. 1I), and of DOX PLGA-CS-PEG was +1.3 mV (Fig. 1J). In addition, the entrapment efficiency (EE%) of DOX for DOX PLGA, DOX PLGA-CS, as well as DOX PLGA-CS-PEG, were 73%, 77%, and 72%, respectively.

### Cytotoxicity of prototypes of PN as well as PNG on colorectal cancer cells

**Fig. 2:**
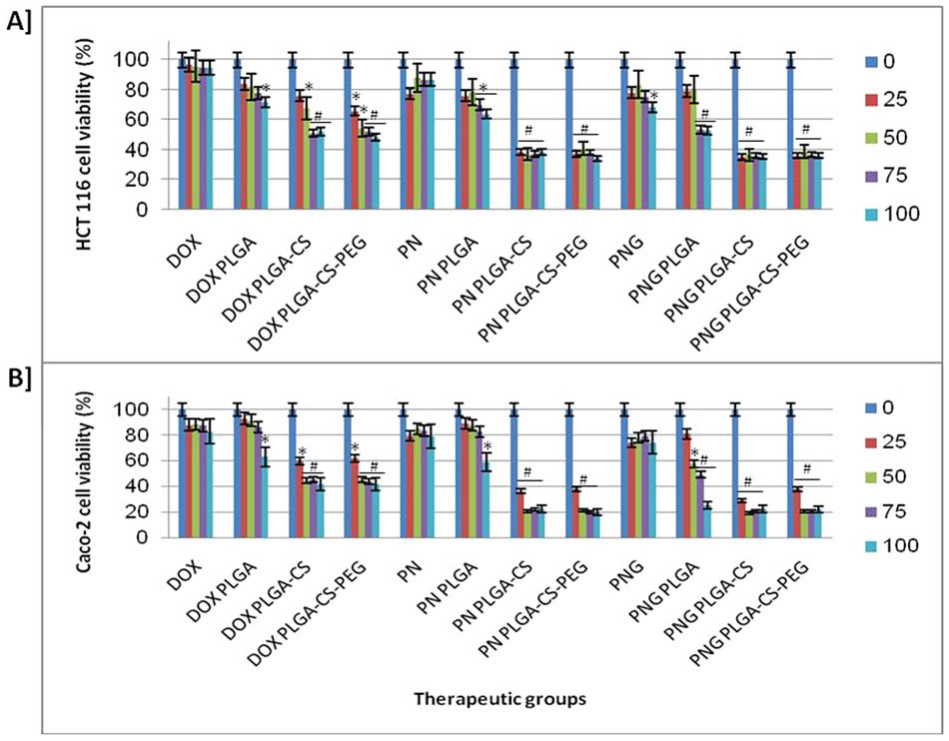
Anti-colorectal cancer activity of the proposed formulations. Cytotoxicity of PN and PNG free- within nano-prototypes in cell lines of HCT 116 (**A**) as well as Caco-2 (**B**) after treatment for 24 h (*n* = 3). * means that there were statistical difference between the tested counterparts and the control (*P* < 0.05). # means that there was high statistical difference between the tested counterparts and the control one (*P* < 0.01). The used concentrations (0, 25, 50, 75, and 100 µM). Zero µM means untreated cells “negative control” and DOX (Doxorubicin) is the positive control

The cytotoxicity of prototypes of the free- as well as nano-PN and PNG versus DOX formulations were investigated in HCT 116 as well as Caco-2 cell lines at varying levels (0, 25, 50, and 75 µM) using MTT assays. Zero concentration refers to the untreated cancerous cells and nominated “control”. The viability of HCT 116 as well as Caco-2 cells after incubation period 24 h treatment by PN and PNG prototypes versus control is shown in Fig. 2. There was significant decrease in cell proliferation of both colorectal cancerous cells (*P* < 0.05) when comparing the treated cells with PN and PNG PLGA NPs but this significance increased dramatically when their PLGA-CS, as well as PLGA-CS-PEG NPs, has been applied (*P* < 0.01).

For the HCT 116 colon cancer cell line, PNG-loaded PLGA-CS-PEG and PN-loaded PLGA-CS-PEG had IC_50_ values of 28.09 µM and 29.5 µM, respectively, indicating significant cytotoxicity compared to unloaded PLGA-CS-PEG (nano-void). In addition, PNG-loaded PLGA-CS and PN-loaded PLGA-CS had potent HCT 116 cell line cytotoxicity (IC_50_ 26.9 µM and 28.01 µM, respectively; Table 1).

**Table 1:** IC_50_ values based on cytotoxic effect of the nanoparticles on colon cancer cell lines

Treatments	HCT 116 cells	Caco-2 cells
DOX	ND	ND
PLGA	ND	ND
PLGA-CS	ND	42.5 ± 4.23^b^
PLGA-CS-PEG	61.203 ± 3.24^a^	43.4 ± 1.24^b^
PN	ND	ND
PN PLGA	ND	ND
PN PLGA-CS	28.01 ± 2.13^b^	22.87 ± 1.35^b^
PN PLGA-CS-PEG	29.5 ± 1.36^b^	23.57 ± 2.01^b^
PGN	ND	ND
PGN PLGA	ND	66.46 ± 3.38^a^
PNG PLGA-CS	26.9 ± 2.24^b^	20.96 ± 0.78^b^
PNG PLGA-CS-PEG	28.09 ± 0.93^b^	23.23 ± 0.83^b^

*DOX* doxorubicin, positive control or reference drug, *PN* punicalin, *PNG* punicalagin, *CS* chitosan, *PLGA* poly-lactide-glycolide, *PEG* polyethylene glycol, *HCT 116; Caco-2* Human colon cancer cell lines, *ND* not detectable (more than 100 µM)

^a^Means that there were statistical difference between the treatments and the reference drug (*P* < 0.05)

^b^Means that there were high statistical difference between the treatments and the reference drug (*P* < 0.01)

For Caco-2 cells, PN and PNG-loaded with PLGA-CS had IC_50_ values of 20.96 µM and 22.87 µM, respectively, indicating significant cytotoxicity in Caco-2 cells compared to unloaded PLGA-CS nanoparticles. Further, PNG and PN-loaded with PLGA-CS-PEG had IC_50_ of 23.23 µM and 23.57 µM, respectively, demonstrating significant cytotoxicity compared to unloaded PLGA-CS-PEG nanoparticles. PNG and PN free counterparts had undetectable IC_50s_ values for both cell lines of colon cancer.

### Cell cycle arrest

**Fig. 3:**
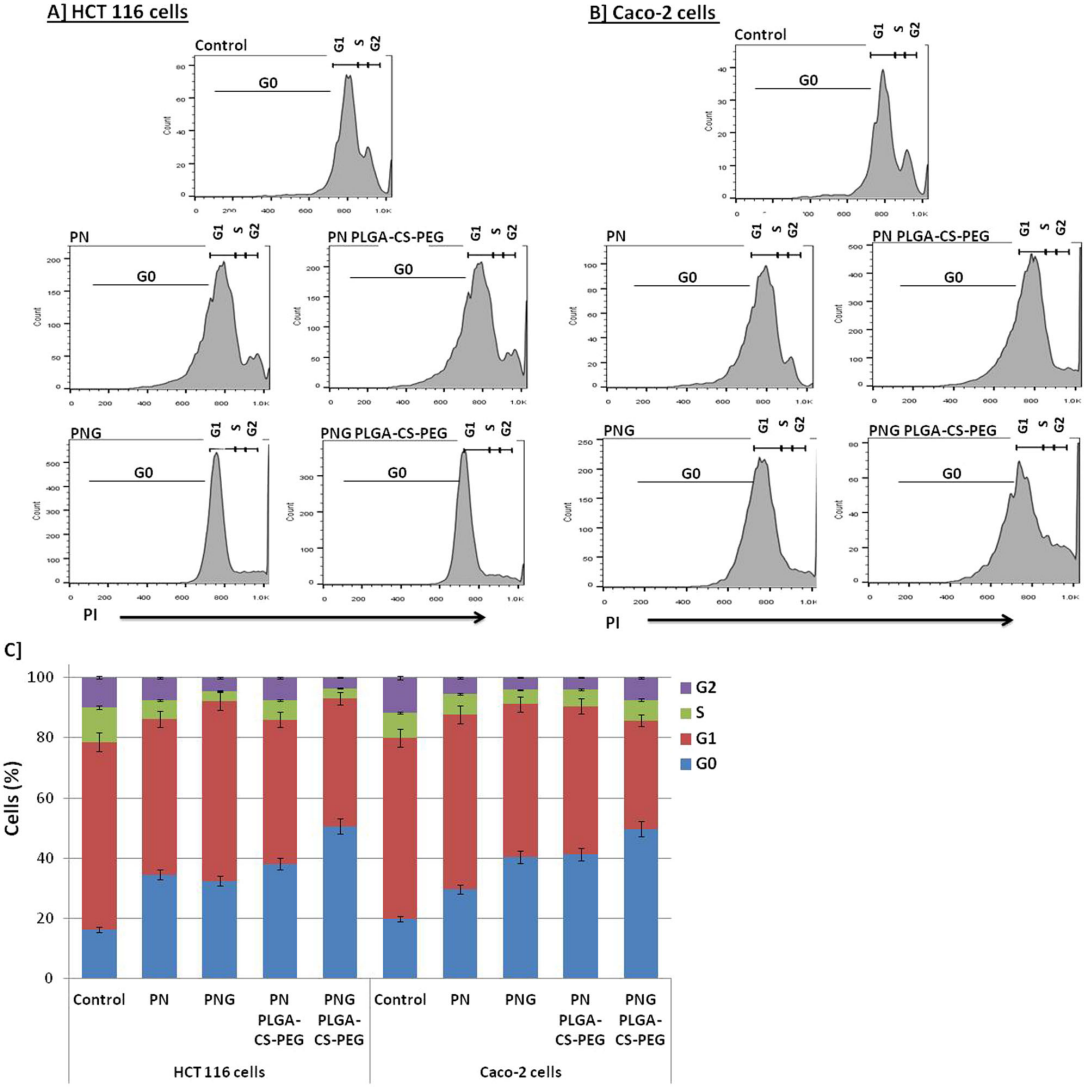
Cell cycle analysis. Cell cycle phases of HCT 116 (**A**) as well as Caco-2 (**B**) cells following treatment within PN and PNG free- and nano-prototypes (using IC_50_ values) for 24 h (*n* = 3). **C** Graph shows the mean and SD values of the cell cycle results (*n* = 3). The statistical analysis was shown clearly in Table 2

For determination of mechanisms of the cytotoxic effects of NPs, the effects of prototypes of PN, as well as PNG on the cell cycle, were investigated. HCT 116 as well as Caco-2 cells have been treated by IC_50_ doses of PN as well as PNG NPs for incubation period 24 h. Finally, a cell cycle distribution analysis was performed on FCM as shown in Fig. 3. The cell cycle phases of HCT 116 (Fig. 3A), as well as Caco-2 (Fig. 3B) cells upon treatment with the proposed PN and PNG free forms and NPs, are shown. A graphical representation providing mean in addition to standard deviation values of the cell cycle results (*n* = 3) is shown in Fig. 3C. Table 2 showed the statistical analysis of the cell cycle phases upon treatments. Upon HCT 116 cell therapy, PN PLGA-CS-PEG showed significance increase at G0 phase and significance decrease at G1and DNA synthesis (S) phases (*P* < 0.05). On the other hand, PNG PLGA-CS-PEG showed high significance increase “i.e., cancer cell accumulation” at G0 phase (*P* < 0.01), significance decrease at G1 phase (*P* < 0.05), and high significance decrease at S and G2 phases (*P* < 0.01). Upon Caco-2 cell therapy, PN PLGA-CS-PEG showed high significance increase at G0 phase (*P* < 0.01), significance decrease at G1 and S phases, and high significance decrease at G2 phase. On the other hand, PNG PLGA-CS-PEG showed high significance increase at G0 phase, high significance decrease at G1 phase, and significance decrease at S and G2 phases.

**Table 2:** Cell cycle analysis of prototypes and nanoparticles on colon cancer cell lines

Cell lines	Treatments	G0	G1	S	G2
HCT 116 cells	Control	16.24 ± 0.81	62.3 ± 3.11	11.7 ± 0.53	9.76 ± 0.23
	PN	34.53 ± 1.7265^a^	51.7 ± 2.5	6.31 ± 0.32^a^	7.46 ± 0.83
	PNG	32.35 ± 1.61^a^	59.7 ± 2.9	3.47 ± 0.32^b^	4.48 ± 1.23^b^
	PN PLGA-CS-PEG	38.07 ± 1.9^a^	48 ± 2.3^a^	6.44 ± 0.72^a^	7.49 ± 1.1
	PNG PLGA-CS-PEG	50.6 ± 2.54^b^	42.5 ± 2.21^a^	3.36 ± 0.16^b^	3.54 ± 0.01^b^
Caco-2 cells	Control	19.78 ± 0.92	60.2 ± 3.2	8.32 ± 0.34	11.7 ± 0.03
	PN	29.59 ± 1.5^a^	58.2 ± 2.91	6.98 ± 0.93	5.41 ± 0.01^b^
	PNG	40.39 ± 1.9^b^	50.8 ± 2.43^a^	4.73 ± 0.23^a^	4.08 ± 0.03^b^
	PN PLGA-CS-PEG	41.17 ± 2.05^b^	49.3 ± 2.42^a^	5.66 ± 0.1^a^	3.87 ± 0.02^b^
	PNG PLGA-CS-PEG	49.67 ± 2.4^b^	36.1 ± 1.81^b^	6.83 ± 0.2^a^	9.76 ± 0.65^a^

*PN* punicalin, *PNG* punicalagin, *CS* chitosan, *PLGA* Poly-lactide-glycolide, *PEG* polyethylene glycol, *HCT 116; Caco-2* Human colon cancer cell lines

^a^Means that there was statistical difference between the treatments and the reference drug (*P* < 0.05)

^b^Means that there was high statistical difference between the treatments and the reference drug (*P* < 0.01)

Colorectal cancer cells accumulated in the G0 phase followed by G1 cell inhibition following treatment with PN and PNG-free forms and these changes became more marked following treatment with nano-formulations of the prototypes of PN along with PNG in both cell lines of colon cancer. Treatment of the HCT 116 cell line with PNG-loaded PLGA-CS-PEG and PN-loaded PLGA-CS-PEG resulted in accumulation of 42.5% and 48.0% of cells in G1 phase, respectively, compared with 62.3% control counterpart. Caco-2 cells Treated with PNG-loaded PLGA-CS-PEG and PN-loaded PLGA-CS-PEG resulted in the accumulation of 36.1% and 49.3% in the G1 phase, respectively, in comparison with control one 60.2%. There was a trend toward a lower proportion of cells in the G1 phase after PN and PNG free forms treatment in both colon cancer cell lines.

There was a substantial decrease in colorectal cancer cells in the S phase after treatment with PN and PNG prototypes. In case of cell line of HCT116, the proportion of cells in the S phase ranged from 11.7% for the control to 3.36% and 6.44% in cells treated with PNG-loaded PLGA-CS-PEG and PN-loaded PLGA-CS-PEG, respectively. In Caco-2 cells, the proportion of cells in the S phase ranged from 8.32% for the control one to 5.66% and 6.83% for cells treated with PN-loaded PLGA-CS-PEG as well as PNG-loaded PLGA-CS-PEG, respectively. Furthermore, there was a trend toward a lower proportion of cells in the S phase following PN and PNG free forms treatment in both colon cancer cell lines.

The proportion of cancer cells in the G2 phase was decreased following treatment with PN and PNG prototypes. In case of cell line of HCT116, the proportion of cells in the G2 phase varied from 9.76% for the control to 3.54% and 7.49% for PNG-loaded PLGA-CS-PEG and PN-loaded PLGA-CS-PEG, treated cells, respectively. In Caco-2 cells, the proportion of cells in the G2 phase ranged from 11.7% for control to 3.87% and 7.4% for cells treated with PN-loaded PLGA-CS-PEG as well as PNG-loaded PLGA-CS-PEG, respectively. Finally, there was a trend toward a lower proportion of cells in the G2 phase following PN and PNG free forms treatment in both colon cancer cell lines.

### Energy blockage of colon cancer cells and induction of stress markers

**Fig. 4:**
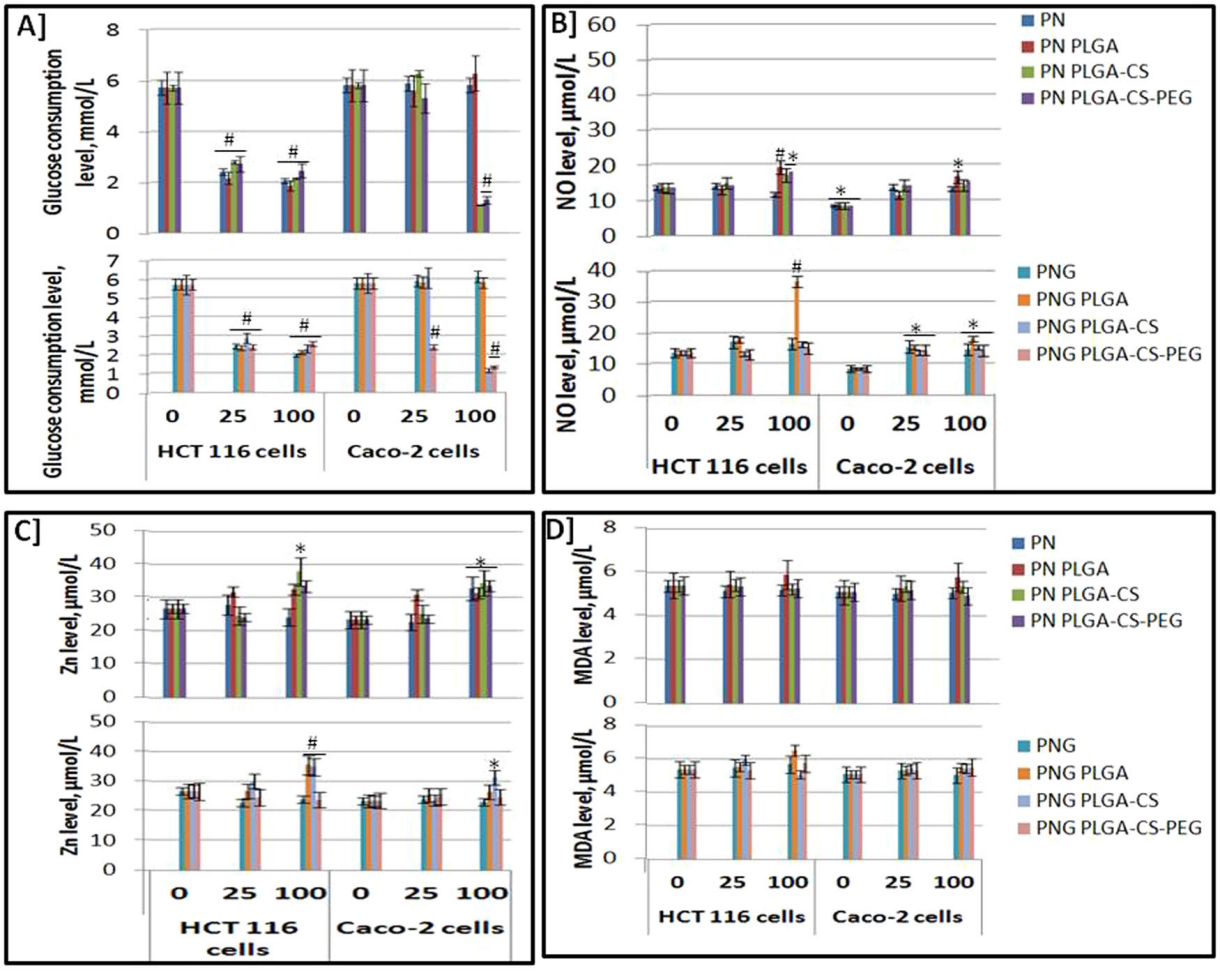
Glucose consumption rate and stress markers. Glucose consumption levels (**A**), NO levels (**B**), Zn levels (**C**), and MDA levels (**D**) in HCT 116 as well as Caco-2 cells following treatment within PN and PNG free- and nano-prototypes (*n* = 3). * means that there was statistical difference between the tested counterparts and the control (*P* < 0.05). # means that there was high statistical difference between the tested counterparts and the control (*P* < 0.01). The used concentrations (0, 25, and 100 µM). Zero µM means untreated cells “negative control”

Figure 4A demonstrates the glucose consumption rate of colorectal cancer cell lines after treatment with 0, 25, and 100 µM of PN as well as PNG NPs in comparison with control counterparts after 24 h incubation. Cellular uptake of glucose from the media of culture significantly elevated with incubation period over 24 h, resulting in a significant diminish in the glucose concentration within culture media (*P* < 0.01).

This findings is likely attributable to inhibition of glucose transporters by nano-formulations leading to decreased glucose transport and glucose consumption following treatment for 24 h. Accordingly, increased glucose levels have been found within media from treated cells compared with the control.

NO (Fig. 4B), Zn (Fig. 4C), and MDA levels (Fig. 4D) were measured in HCT 116 as well as Caco-2 cells after treatment with different PN and PNG nano-prototypes. NO concentration has been increased significantly (*P* < 0.05) after nano-PN and nano-PNG treatment compared to the control, while insignificant changes differences in MDA levels have been found between nano-prototypes as well as control. In addition, Zn content has been increased significantly following treatment with PN as well as PNG (Fig. 4C). This finding demonstrates that prototypes of PN as well as PNG react with Zn to promote the death colorectal cancer cells; however, the identical mechanism of action has yet to be revealed.

### Genetic and protein profiles

**Fig. 5:**
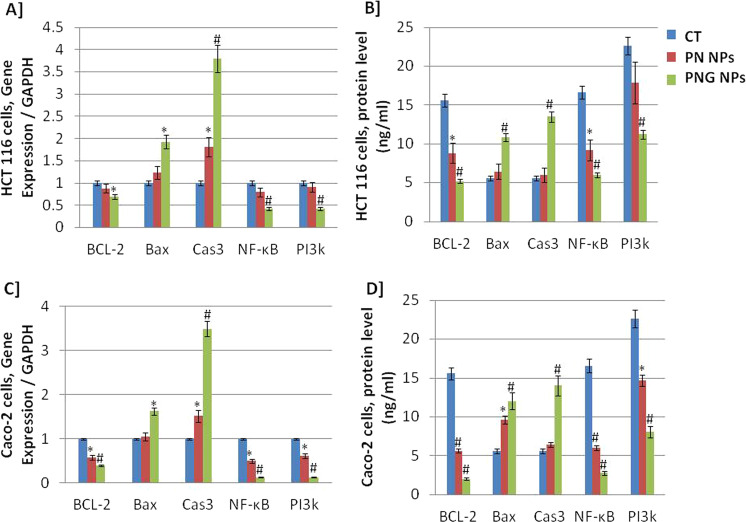
Genetic and proteomic analyses. Real-time qRT-PCR and ELISA measured expression and protein levels of BCL-2, Bax, Cas3, NF-kβ, and PI3k in HCT 116 (**A**, **B**) as well as Caco-2 (**C**, **D**) cells following treatment with free- and nano-PNG and PN for 24 h using IC_50_ values. * means that there was statistical difference between the tested groups and the control (*P* < 0.05). # means that there were high statistical difference between the tested groups and the control (*P* < 0.01)

Genetic and protein expression levels of BCL-2, Bax, Cas3, NF-kβ, and PI3k were assessed in HCT 116 (Fig. 5A, B) as well as Caco-2 (Fig. 5C, D) cells after treatment with nano-PN and nano-PNG nanoparticles. The IC_50_ dosages were used with a 24-h incubation period.

Treatment with PNG nanoparticles resulted in marked up-regulated expression and increased protein levels significantly of markers of apoptosis-like BAX and Caspase-3 (Cas3) in HCT 116 as well as Caco-2 cells compared with treatment with PN NPs and control. In addition, treatment with PNG nanoparticles resulted in significant down-regulation of BCL2, PI3k, and NF-κB in HCT 116 as well as Caco-2 cells in comparison to treatment with PN NPs and control.

## Discussion

The WHO has highlighted colon cancer as a particular concern due to increasing rates in adolescent, young adult, and adult populations. Further, the WHO has reported that CRC remains one of the leading causes of cancer-related deaths globally [13]. Conventional synthetic drugs may cause severe systemic toxicity and long-term administration is associated with dangerous side effects. Further, tumor resistance and recurrence are commonly observed in cases of CRC. Accordingly, many clinical and preclinical studies have aimed to identify novel chemoprevention agents from natural products with high selectivity toward cancer cells with minimum side effects during long-term use [14]. Nanotechnology has been used to modify the pharmacokinetics and reduce systemic toxicity of novel anticancer therapies by targeting therapies to tumor tissues [5, 11].

The incorporation of PLGA, CS, and PEG into our nano-carrier provides novel advantages in PN and PNG delivery. Poly (lactic-co-glycolic acid) nanoparticles (PLGA NPs) represent an idealistic model of drug delivery carriers due to their biocompatibility and biodegradability. Chitosan (CS) was used to coat PLGA nanoparticles due to its cationic charge, biodegradability, and mucoadhesive properties. Additionally, PEG exhibits minimum toxicity and increases the penetrating potential of molecules across mucosal surfaces. Stearic acid-grafted CS oligosaccharide micelles represent a efficient candidate as drug delivery carriers intracellularly because of their specific spatial structures [15, 16].

Within the present study, PLGA-CS-PEG NPs were used to rapidly deliver, diminish clearance, and enhance the cancer-targeting ability of PNG and PN to cancer cells. Accordingly, PNG-PLGA-CS-PEG and PN-PLGA-CS-PEG NPs were found to have very low IC_50_ values (28.09 µM and 29.5 µM, respectively) and high cytotoxicity against HCT 116 cells. Further, PNG-PLGA-CS-PEG and PN-PLGA-CS-PEG NPs had even lower IC_50_ values (20.96 µM and 22.87 µM, respectively) against Caco-2 cells compared to other proposed formulas. Thus, Caco-2 cancer cells appear to extra sensitive to NPs than HCT 116 cancer cells. These results corroborate our recently published data applied human cell lines of breast cancer [5] demonstrating that PNG NPs have an IC_50_ of 34.6 µM against MCF7 cancer cells, while PN NPs have an IC_50_ of 30.9 µM against breast cancer cells (MDA-MB-231). Thus, human breast cancer cells (MDA-MB-231) are more sensitive to NPs than human colon MCF7 cancer cells.

PNG and PN can be extracted in the form of an ellagitannin structure from numerous natural plant materials including juice from pomegranates, their fruits, seeds, blossoms, leaves, and bark, as well as myrobalan (Terminalia chebula Retz.), yellow wood (Terminalia oblongata F. Muell.), tropical almond, and their pericarp (peel) (Terminalia catappa L.) [17, 18]. The anticancer activity of PNG as well as PN was revealed in numerous cell lines of cancer. PN and PNG possess the ability to modulate pathways of signal transduction related to proliferation and survival to promote the arresting of the process of cell cycle, autophagy, senescence and apoptosis. The PI3K/Akt/NF-kβ signaling acts an essential role in cancer therapy through regulation of cell proliferation, apoptosis, and angiogenesis and alterations in this pathway are significant contributors to tumor drug resistance [19]. PNG and PN may slow cancer progression and metastasis by modulating the PI3K/Akt/NF-kβ signaling pathway. PNG and PN are posited to suppress phosphorylation of PI3K/AKT and mTOR expression, indicating modulatory effects on the mTOR pathway [19]. PGN and PN have been shown to induce apoptosis via stimulation of the pathway of intrinsic-mitochondrial leading to the libration of apoptotic signals via caspase-3/-8/-9 and Bax stimulation and the induction of cytochrome C as well as suppression of Bcl-XL and Bcl-2 expression. In addition, PN and PNG influence NF-κB signaling through the active phosphorylated form of IκBα prevention and inhibition of translocation of NF-κB-p65 to the cancer cells’ nucleus [3, 17, 20, 21]. Moreover, Berkoz et al. [18] and Abd-Rabou et al. [5] reported that both of PNG and PN have substantial antioxidant activities. Polyphenols have been reported to increased the uptake of zinc in addition to expression of caco-2 cells metallothionein (MT). The expression of MT plays an essential role in trafficking of zinc extracellularly and intracellularly [22, 23]. High cellular zinc levels may promote the process of Bax-associated mitochondrial pore-forming that activates cytochrome c libration leading to the initiation of the caspase cascade to induce apoptosis [24, 25]. This assumption has confirmed by the findings of our current work that demonstrate PN and PNG NPs induce ROS-mediated cell death through cell cycle arrest and suppression of PI3K/NF-kβ pathway.

PNG reportedly induces arresting of cell cycle in the G0/G1 phase, thereby blocking the proliferation of the cell. However, PNG appears to have no effect on ROS overproduction, although PNG significantly induced membrane potential loss leading to cytotoxicity. PNG reportedly had no effect on redox homeostasis leading to increased cell death in yeast cells, likely due to the properties of PNG as a ROS scavenger [26]. Some of these results corroborate the findings of the present study where our PNG NPs motivated arresting of the cell cycle at G0/G1 phase, thereby cell proliferation prevention accompanied by promoting cell death; however, our nano-formula caused significant ROS overproduction and alterations in redox homeostasis to promote cell death in colorectal cancer cells. Interestingly, previous studies have shown natural polyphenols can induce ROS-dependent and ROS-independent cell cycle arrest in cancer cells [27].

## Conclusions

Ultimately, the current work data demonstrated the therapeutic efficacy of punicalagin (PNG) and punicalin (PN) NPs in HCT 116 as well as Caco-2 cell lines. PNG and PN NPs may have utility in the treatment of colon cancer through the induction of ROS-mediated cell cycle arrest and suppression of the signaling pathway of PI3K/NF-kβ.

## Data Availability

Available after authors’ approval.
